# Re-examining advice to complete antibiotic courses: a qualitative study with clinicians and patients

**DOI:** 10.3399/BJGPO.2022.0170

**Published:** 2023-03-08

**Authors:** Aleksandra J Borek, George Edwards, Marta Santillo, Marta Wanat, Margaret Glogowska, Christopher C Butler, Ann Sarah Walker, Gail Hayward, Sarah Tonkin-Crine

**Affiliations:** 1 Nuffield Department of Primary Care Health Sciences, University of Oxford, Oxford, UK; 2 National Institute for Health and Care Research (NIHR) Health Protection Research Unit in Healthcare Associated Infections and Antimicrobial Resistance, University of Oxford, Oxford, UK; 3 Nuffield Department of Medicine, University of Oxford, Oxford, UK; 4 NIHR Oxford Biomedical Research Centre, Oxford, UK

**Keywords:** antibiotic course, drug resistance, microbial, antimicrobial stewardship, communicable diseases, primary health care, qualitative research

## Abstract

**Background:**

Antibiotic treatment duration may be longer than sometimes needed. Stopping antibiotics early, rather than completing pre-set antibiotic courses, may help reduce unnecessary exposure to antibiotics and antimicrobial resistance (AMR).

**Aim:**

To identify clinicians' and patients' views on stopping antibiotics when better (SAWB) for urinary tract infections (UTIs), and to explore comparisons with other acute infections.

**Design & setting:**

An exploratory qualitative study with general practice clinicians and patients in England.

**Method:**

Primary care clinicians and patients who had recent UTI experience were recruited in England. Remote one-to-one interviews with clinicians and patients, and one focus group with patients, were conducted. Data were audiorecorded, transcribed, and analysed thematically.

**Results:**

Eleven clinicians (seven GPs) and 19 patients (14 with experience of recurrent and/or chronic UTIs) were included. All participants considered SAWB unfamiliar and contradictory to well-known advice to complete antibiotic courses, but were interested in the evidence for risks and benefits of SAWB. Clinicians were amenable if evidence and guidelines supported it, whereas patients were more averse because of concerns about the risk of UTI recurrence and/or complications and AMR. Participants viewed SAWB as potentially more appropriate for longer antibiotic courses and other infections (with longer courses and lower risk of recurrence and/or complications). Participants stressed the need for unambiguous advice and SAWB as part of shared decision making and personalised advice.

**Conclusion:**

Patients were less accepting of SAWB, whereas clinicians were more amenable to it. Patients and clinicians require good evidence that this novel approach to self-determining antibiotic duration is safe and beneficial. If evidence based, SAWB should be offered with an explanation of why the advice differs from the ‘complete the course’ instruction, and a clear indication of when exactly to stop antibiotics should be given.

## How this fits in

Stopping antibiotics when feeling better (rather than completing the full prescribed course irrespective of recovery) could reduce exposure to antibiotics and risks of AMR, but the attitudes of clinicians and patients to the novel approach are unknown. Patients, especially those with more severe or recurrent and/or complicated UTIs, were concerned that SAWB would lead to complications and thus were more averse to it. Clinicians were more amenable to SAWB if the advice was supported by guidelines and some already gave the SAWB instruction with longer antibiotic courses. Clinicians and patients require good evidence that self-determining antibiotic duration is safe and beneficial before they would be comfortable implementing it. If evidence supports this approach, patients need to be offered a clear explanation as to why the advice is different from the previous dogma of 'complete the course'. They also need to be given unambiguous advice on when exactly to stop antibiotics (that is, at which point of symptom improvement or after symptoms resolve).

## Introduction

The perceived wisdom is that all antibiotic courses should be completed because this will limit risk of relapse and selection of resistant organisms. The use of and exposure to antibiotics is a major contributor to AMR,^1^ impacting populations over the long term and individuals in the short term. Patients prescribed an antibiotic have been shown to have a higher risk of experiencing resistance to it up to 1 year after treatment, and longer durations and multiple courses have been associated with higher rates of resistance.^2^ Using an antibiotic from one class has been associated with resistance to another.^3^ Moreover, patients with resistant infections have been more likely to experience slower recovery and to re-consult with GPs, adding burden for patients and clinicians.^4,5^

Different strategies have been implemented in England, which have helped to reduce overall antibiotic prescribing in general practice.^6^ However, further progress could be achieved by focusing efforts on optimising the duration of antibiotic courses and/or stopping antibiotics once patients feel recovered rather than completing the full courses, regardless of recovery. This is provided it does not lead to patient harm or drive resistance. It is often thought that AMR is caused by stopping antibiotic treatment before achieving full cure because untreated and resistant pathogens re-emerge (that is, ‘target selection’).^7^ This view historically supported longer antibiotic courses and the advice to complete them. However, arguably this is not the case for infections usually treated in primary care, which are caused by pathogens commonly (and often harmlessly) occurring in or on our bodies, and where patients’ immune systems are largely unimpaired. For these infections, AMR develops through ‘collateral selection’ and its risk increases the longer one is exposed to antibiotics.^7^ Thus, reducing antibiotic exposure (‘shorter is better’) could help limit AMR.^7,8^

For common infections, many antibiotics have been prescribed for longer than the courses recommended in guidelines; consequently, one way to reduce antibiotic use and exposure could involve better aligning prescribed durations with guidelines.^9^ Moreover, duration of antibiotic courses may be longer than needed and not always evidence based.^7,8,10^ Increasingly, clinical trials have shown that shorter courses are just as effective as longer ones.^8^ Even single doses of some antibiotics (for example, fosfomycin) could be effective in certain contexts.^11,12^ A recent addendum to the UK national action plan for tackling AMR has highlighted the need for clinical research comparing antibiotic course lengths and their impact on patients and AMR.^13^ Therefore, a second possible way to reduce antibiotic exposure and use could involve shortening the recommended antibiotic courses. A third option is to recommend that patients stop antibiotics when they feel better, as illnesses vary considerably and individuals might respond differently to antibiotics and course lengths.^7^ For many infections in people who are not immunocompromised, once recovery is established, relapse is rare regardless of antibiotic treatment.^7^

This study explored views about shorter antibiotic courses and the (hypothetical) advice to SAWB (stopping antibiotics when better). The study focused on UTIs for a number of reasons. First, consultations for symptoms attributed to UTIs are common and are usually (up to 93% of cases) treated with antibiotics in primary care.^14,15^ There appears a clinically unwarranted variation in management of uncomplicated UTIs, without evidence of differences in recovery times.^15^ The study did not focus on other infections because immediate antibiotics are not recommended for most other common infections in generally healthy adults (when avoiding or delaying antibiotics is preferred), making the SAWB approach less relevant. Second, the recommended antibiotic course in the UK has been shortened from 5 to 3 days for lower UTIs in non-pregnant females,^16^ but better evidence for optimal treatment approaches is needed (for example, regarding the course length, dose, and SAWB).^13^ Third, in England only 63% of females prescribed antibiotics for UTIs reported taking them, suggesting that UTIs may resolve with fewer or no antibiotics.^14^ A recent meta-ethnography of qualitative studies on experiences of UTIs has shown that antibiotics are perceived both as a valuable treatment and as a last resort.^17^ Finally, UTIs often resolve rapidly on starting antibiotics so SAWB might be potentially suitable. To broaden the scope beyond UTI alone, the interviews explored also the perceived applicability to, and comparisons with, other acute infections in primary care.

The study aimed to explore the perceptions of primary care clinicians and patients who experienced UTI symptoms to understand their views of shorter antibiotic courses, SAWB, and what would be required for a potential change to be acceptable.

## Method

### Recruitment and sampling

General practice clinicians and patients were recruited in England. Clinicians were included if they managed UTIs in general practice. They were recruited through professional networks and research team contacts. Patients were included if they self-reported experiencing at least one episode of UTI symptoms in the past year. They were recruited through online advertisements, social media, and UTI-related patient groups. The study aimed to include diverse samples of clinicians (based on clinical roles and experience) and patients (based on age, sex, and number of UTIs). Potential participants were asked to contact the researchers and were provided with study information. All participants gave consent verbally, with written records retained. Participants (or clinicians’ practices) were reimbursed for their time.

### Data collection

Patients could choose to participate in an interview or focus group. Clinicians were interviewed individually. Data were collected remotely using telephone or video calls. Two researchers collected data using semi-structured topic guides (see Supplementary Appendix S1). Clinicians were asked about their experiences of managing UTIs, advising patients on UTIs and antibiotics, and views on SAWB. Patients were asked about their experiences of consulting for and managing UTIs, beliefs about antibiotics and AMR, and views on SAWB. Interviews and the focus group were audiorecorded, transcribed verbatim, and transcripts were anonymised. Most data were collected in June 2021 and analysed subsequently. A few additional interviews were then conducted in February–March 2022 (with participants recruited in the same way) to further explore and refine the themes, and ensure that the themes were well-supported by data.

### Analysis

The key points and views discussed by each participant were summarised to inform the analysis, interpretation, and comparisons between different participants. A ‘codebook-type’, pragmatic, realist approach to thematic analysis was used.^18,19^ Four experienced qualitative researchers each inductively coded two clinician and two patient transcripts using NVivo (version 12) software, and identified candidate categories by grouping related codes together. Codes and categories identified by each researcher were compared and discussed, and common categories were agreed. The researchers’ NVivo files were merged and reviewed, resulting in two hierarchical codebooks: one for clinician and one for patient data. The remaining data were coded using the agreed codebooks. The codes and categories, and data summaries, were compared to identify similarities and differences between clinician and patient views, and between participants with different characteristics (for example, professional roles and UTI experiences). Themes were identified and agreed on through team discussions of the data and the codebooks.

## Results

Eleven clinicians and 19 patients participated (three patients participated in one focus group). Sample characteristics are in Table 1 and in Supplementary Boxes S1 and S2.

**Table 1.** Sample characteristics

Characteristics	*n* ^a^
**Clinicians (*n* = 11)**	
Interview length, minutes, range (mean)	29–55 (43)
Professional role	
GP	7
Advanced nurse practitioner	2
Clinical pharmacist	1
Physician associate	1
Years in the current role, range (median)	2.5–40 (6)
Years of clinical experience, range (median)	8.5–47 (16)
Sex	
Female	7
Male	4
**Patients (*n* = 19)**	
Focus group length, minutes	77
Interview length, minutes, range (mean)	25–51 (39)
Sex	
Female	18
Male	1
Age, years, range (median)	21–81 (43)
Self-reported experience of UTIs^b^	
Females with recurrent and/or chronic UTIs (currently or at some point in life)	14
Females with 1–3 uncomplicated UTIs in the past year (without history of recurrent and/or chronic UTIs)	4
Male who experienced one UTI	1

^a^Unless otherwise stated. ^b^Supplementary Boxes S1 and S2 contain a brief summary of each patient's experience of UTIs. UTIs = urinary tract infections.

All participants expressed both positive and negative views (that is, reasons to be amenable and/or open and concerned and/or averse) about SAWB (Table 2). Overall, clinicians seemed more amenable to SAWB than patients, but a clear pattern of different views depending on clinicians’ roles was not found. Patients with recurrent and/or chronic UTIs seemed more averse to SAWB than patients with less severe experiences. Each participant's main views are summarised, with their roles and experiences, in Supplementary Boxes S1 and S2.

**Table 2.** Main arguments about SAWB advice

Themes	Reasons to be amenable to the SAWB advice	Reasons to be concerned or sceptical about the SAWB advice
Change in evidence, guidelines, and education required for SAWB	Medicine evolves (antibiotic courses are not always evidence based and have shortened), so open to SAWB if evidence and guidelines change (C) Antibiotic courses seem arbitrary — clinicians prescribe different courses and many patients do not take full courses anyway (C)	SAWB is unfamiliar and at odds with the ingrained advice to complete antibiotic courses (C and P)
Current approach to antibiotics influences attitudes to SAWB	SAWB seen as more appropriate and beneficial with longer antibiotic courses for UTIs or other infections (C and P), especially as it is already given with longer courses (C) SAWB advice already given or used in recurrent UTIs (C and P)	3-day antibiotics for UTIs are already short so SAWB would be less or not relevant for UTIs (C and P) SAWB with short courses for UTIs would have little impact or benefit so it is not a priority (C) Patients with experience of recurrent and/or complicated UTIs perceived current courses as already too short so were against stopping even earlier (P)
Balancing risks and benefits of SAWB is needed	Would consider SAWB if evidence shows it is safe and beneficial (C and P) SAWB may help reduce antibiotic side effects (C and P) and antimicrobial resistance (C) SAWB may be more suitable for other infections than UTIs where risks of recurrence and/or complications are lower (P)	SAWB may lead to recurrence, complications, and antimicrobial resistance (C and P) Participants with recurrent and/or chronic UTIs were particularly concerned about SAWB causing resistant UTIs (P)
Importance of effective communication and personalisation of SAWB	SAWB may help empower patients as part of shared decision making (C and P) SAWB advice more acceptable from a trusted clinician and when personalised (P)	Unsure how SAWB should be best formulated and that it may be unclear to patients when to stop antibiotics (C and P) Unsure and concerned about what happens with unused or leftover antibiotics (C and P) SAWB inappropriate for those perceived to be unable to make treatment decisions (C and P)

C = views expressed by clinicians. P = views expressed by patients. SAWB = stopping antibiotics when better. UTI = urinary tract infection.

The key findings were organised into four themes, illustrated with selected quotes.

Throughout, references to ‘participants’ mean both clinicians’ and patients’ views; otherwise it is stated that the reported view was expressed by participating ‘clinicians’ or ‘patients’ only.

### Change in evidence, guidelines, and education required for SAWB

Participants found the SAWB advice unfamiliar and contradicting the ‘*ingrained*’ advice to complete antibiotic courses to prevent AMR. Patients in particular expressed surprise and scepticism about SAWB.

*'It’s ingrained into the psyche of both patient and doctor that you complete your course. It’s ingrained in such a way that it is going to take something quite substantial to shift that.'* (GP-5)

*'I wouldn’t like* [SAWB] *— that would make me question them quite a lot as a doctor. Because it’s always been drummed into me that you should absolutely always finish your course, because not finishing it contributes to antibiotic resistance … '* (Patient-15, multiple UTIs)

Participants considered SAWB to be *‘quite a big turnaround’* (Patient-1) that would require *‘undoing that bit of education and re-explaining’* (GP-6) and wider promotion (for example, public health campaigns). Participants wanted the reasons for changing the advice or approach to be explained to them.

Nevertheless, some clinicians expressed openness to SAWB as they considered medicine, evidence, and guidelines as ‘evolving’. They questioned how evidence based the current antibiotic courses were and noted that many courses had already been shortened. They stated that if the evidence and guidelines changed to support SAWB, they would adopt it:

*'I’m receptive.* […] *medicine … constantly evolves.* […] *I pick up currents and I let that sort of sway my practice. I’m aware there’s a bit of trend towards shorter courses … '* (GP-7)

Some clinicians’ openness to SAWB seemed also linked to a perceived variation in how other clinicians prescribe, and to a perception that many patients did not take full courses anyway:

*'… some doctors will give antibiotics* […] *for 5 days, some for 7 days, some for 10 days. It seems pretty arbitrary sometimes.* […] *I know that plenty of people don’t finish their course of antibiotics so if someone’s asking my permission to do that, absolutely I’ll tell them that it’s not a problem as long as they’re feeling better.'* (GP-3)

### Current approach to antibiotics influences attitudes to SAWB

SAWB was considered within the context of managing UTIs and prescribing or using antibiotics. Participants described antibiotics as the default, expected treatment for UTIs. Clinicians described *‘the drive to give antibiotics* [as] *higher’* (GP-3) for UTIs because of bacterial aetiology, compared with respiratory infections, which are more likely viral. Most patients described urgently wanting antibiotics (*‘the solution right away’* [Patient-13] and *‘a hefty dosage’* [Focus group {FG}-Patient-1]) to quickly resolve painful and disruptive UTI symptoms and prevent longer-term problems. Only a few patients reported that when experiencing milder symptoms, and depending on personal circumstances, they tried self-managing UTIs before consulting for antibiotics.

Participants reported typically prescribing or receiving 3-day antibiotic courses for females with UTIs. They considered this course as already short, with UTIs taking a couple of days of antibiotics to improve, and perceived the SAWB advice as less or not impactful or appropriate. Clinicians perceived SAWB as a small saving (*‘a marginal gain’* [GP-3]) and not a priority for short courses. Patients were concerned that the 3-day course was already *'too short'* to fully resolve UTIs. Some participants viewed SAWB as potentially more beneficial for other infections with typically longer courses:

*'It’s going to take a day or two for them to feel better anyway, so it’s not such a big deal. For me that’s not really a priority to even discuss finishing that* [a 3-day course] *early, but if you’re talking about a week’s course of antibiotics for a chest infection and they’re feeling better after 4 days, then you’re saving a significant number of days of antibiotics and I would tell them to stop.'* (GP-3)

Some participants perceived SAWB as more beneficial and appropriate with longer antibiotic courses for UTIs (for example, 5–7 days and SAWB at or after 3 days). Some clinicians reported already giving the SAWB advice when prescribing an *'extended or longer'* antibiotic course and/or when guidelines indicated a range of durations (for example, *‘7–14 for bronchiectasis’* [Advanced nurse practitioner {ANP}-2]).

*'… occasionally we do give longer courses of antibiotics because the five days isn’t enough so we’ll sometimes say we’ll give you a longer course but we would recommend that you stop it at five days if your symptoms have cleared.'* (Physician Associate)

A few participants reported also giving or following the SAWB advice for frequent, recurrent UTIs when patients have antibiotics ‘on standby’ to use when UTI symptoms appear and stop after they resolve.

*'I have antibiotics on repeat prescription, and an agreement with my GP that I can keep them* […] *and when I’m confident that I have a UTI, I will choose to start those, and there is no set duration of treatment, and I’ll sort of play it by ear and continue taking the antibiotics for 48 hours after my symptoms have stopped.'* (Patient-16, recurrent UTIs)

### Balancing risks and benefits of SAWB is needed

Participants were concerned about risks of UTI recurrence and/or complications and AMR. They perceived completing full courses as preventing AMR, whereas stopping antibiotics earlier (for example, SAWB or shorter courses) as contributing to recurrent UTIs and AMR. Patients with experience of recurrent and/or chronic UTIs felt particularly strongly about this and against SAWB advice.

*'… if I’m giving somebody 3 days’ antibiotics* […] *the antibiotic hasn’t had enough time to work on their bacteria and their infection would come back because it hasn’t resolved. So my thinking would be complete the course of antibiotics and then there’s less likely antimicrobial resistance in the long term* ... *'* (ANP-2)

*'I don’t think that* [SAWB] *is appropriate advice* […] *The main concern for me is the development of resistant bacteria and the development of a long-term life-limiting condition because that’s exactly what happened to me*.*'* (Patient-6, chronic UTI)

Nevertheless, participants also described benefits of taking fewer antibiotics, mostly in reducing side effects. Patients expressed reluctance to take antibiotics because of side effects, the impact on the microbiome and gut, or perceived overmedication. For some, this meant they would be open to SAWB (if it was safe):

*'… if your symptoms have gone, if you feel better, why take a tablet?'* (Patient-1, one recent UTI and history of resistant UTI)

However, for UTIs, most perceived it as a necessary *‘trade-off’* (FG-Patient-1) between side effects and the need to treat UTIs:

*'I don’t like taking* [antibiotics]*, in general, because of the whole resistance* […] *sometimes they can make me feel quite ill and sick* […] *but then with UTI, because they’re so severe and horrible, I feel like they’re necessary sometimes*.*'* (Patient-5, multiple UTIs)

Participants wanted to know the risks and benefits of the different approaches to managing UTIs and SAWB, whether it is safe and beneficial. Clinicians were particularly interested in evidence of effectiveness of SAWB and its impact on AMR. Patients were interested in risks and benefits of one longer course of antibiotics compared with multiple shorter courses.

*'I don’t know that I know enough about* [SAWB] […] *Is it an effective strategy and does it actually achieve something meaningful?* […] *If it’s a little bit less effective in terms of relapse rates but does substantially* [reduce] *resistance rates, that could be a trade-off worth having.'* (GP-7)

*'The benefit of potential reduction in AMR — which we don’t actually know if it would achieve that or not, or if it would increase it — versus the risk of women increasingly getting recurrent UTIs which, for some women, could be really unlucky and it leads to embedded UTIs — is just not worth the risk with the evidence that we currently have available.'* (Patient-8, chronic UTI)

Many patients reported that they would consider SAWB if there was a reliable test to *‘categorically’* show whether an infection has fully cleared before stopping antibiotics (the current tests were perceived as unreliable):

*'If you could* […] *have a test and then have a test two days later, and then they could tell you categorically that* [the UTI] *had gone, that would be different but at the moment it’s really like roulette*.*'* (FG-Patient-2, chronic UTI)

Finally, some patients perceived SAWB as more suitable for other acute infections when the risks of recurrence were seen as lower than in UTIs:

*'… infection in an eye or in a leg or anything like that, when you stop your antibiotics it doesn’t tend to come back, that tends to go, so it’s a very different thing* [to UTIs].*'* (Patient-10, recurrent UTIs)

### Importance of effective communication and personalisation of SAWB

Clinicians expressed mixed views on what SAWB might mean: whether patients should stop antibiotics when symptoms improved or resolved, and wanted guidance on this area. In contrast, patients thought that the advice should only refer to stopping antibiotics when UTI symptoms fully resolved and, for some, symptoms should be absent for 24 or 48 hours before stopping antibiotics:

*'I’d probably want to have some guidance on that.* […] *I’m making that up off the top of my head. I’d be looking at what symptoms they’re predominantly experiencing and sort of looking for either resolution or I guess 90% resolution of symptoms.* […] *I’m not imagining that everything would be absolutely 100% but substantial improvement.'* (GP-7)

*'Feeling well, back to normal.* […] *all pain and all symptoms have gone, and that they’ve been gone for full 24–48 hours. I’d actually say 48 hours. Once you’ve had two full days of no symptoms possibly stop the antibiotics, but certainly not before that.'* (Patient-10, chronic UTI)

Moreover, patients were concerned that it might be unclear when exactly to stop antibiotics, relying too much on *‘subjective’* (FG-Patient-3) feeling and leading to *‘overthink*[ing]*’* symptoms:

*'… I would then turn it in my head, "Do I feel better?", I can imagine myself being like “Are the symptoms totally gone?”* […] *I tend to overthink things, and doubt myself.'* (Patient-14, 1 UTI)

Similarly, patients were unsure about knowing when to stop antibiotics for other infections with symptoms (for example, cough) that could last longer and gradually improve.

Some clinicians considered the value of the SAWB advice in empowering patients to have more autonomy over decisions and as part of shared decision making:

*'*[SAWB] *is just giving* [patients] *more autonomy really. It’s informing and educating them, it’s being less paternalistic* […] *it’s empowering them to make some decisions about their health ...'* (GP-3)

Some patients discussed the importance of trust, continuity of care, and personalised care (for example, tailoring the approach to the patient’s experience and context), and considered SAWB as more acceptable if given in such a context or way. Patients with recurrent and/or chronic UTIs considered themselves knowledgeable about UTIs and confident to actively participate in the treatment and/or management decisions, more so for UTIs than for other infections that they rarely experienced:

*'… with a UTI, I’m so familiar, I feel very confident in managing it. If it was something I hadn’t had routinely, I would be guided by whatever the clinician had said in terms of how to manage the course. Trusting that they would be working from best current practice*.*'* (Patient-16, multiple UTIs)

However, participants also thought that some patients may not want to, or be able to, make decisions when to stop antibiotics, so the SAWB advice would be inappropriate for some (for example, older adults with infection-related confusion).

Overall, participants identified questions that should be addressed, and suggestions, if the SAWB advice was to be implemented (Box 1).

Box 1 **Questions and suggestions identified by participants posing implications for research and practice**

- What is the evidence on different antibiotic course lengths, risks and benefits related to complications and AMR, and for whom the SAWB advice would be appropriate or not? Especially:
  - the impact of shorter courses on risks of recurrence and complications;
  - the impact of multiple shorter versus one longer course on AMR;
  - for which patients with UTI symptoms SAWB is or is not safe;
  - for which other infections that may benefit from antibiotics SAWB is or is not safe.
- What is the evidence on when exactly UTIs are sufficiently ‘treated’ to prevent recurrence? When is it safe to stop antibiotics? For example, should antibiotics be stopped:
  - when symptoms improve (one feels better), and if so, which symptoms and how exactly to define improvement;
  - when symptoms fully resolve, immediately or at a certain point (for example, 24 or 48 hours) after symptom resolution;
  - when a diagnostic test indicates no infection.
  - How the point at which it is safe to stop antibiotics might be different for different infections?
- Need to improve the public or patient and clinician understanding of resistance and UTIs:
  - how AMR develops (in general and in different infections; for example, through target or collateral selection);
  - how to best prevent and manage UTIs to minimise risks of long-term problems (recurrent and chronic UTIs).
- Need for better communication and personalisation of the SAWB approach to patient history, concerns (for example, about risks), and views (for example, about antibiotics), including:
  - explaining what the SAWB approach is and reasons for it.
- Need for more reliable diagnostic tests to inform UTI management, for example:
  - to reliably determine whether a UTI is present or not (and whether an antibiotic is needed);
  - to reliably determine when a UTI is cleared (treated) before stopping antibiotics.
- Need for better non-antibiotic treatments to prevent and manage UTIs.

## Discussion

### Summary

‘Finish the course’ is a well-known and widely accepted instruction regarding antibiotic use. However, in primary care, this approach is inadequately supported by clinical evidence, whereas SAWB may help reduce unnecessary exposure to antibiotics and AMR. The study found that clinicians were more amenable to SAWB than patients, mostly because they acknowledged that evidence changes, reducing antibiotic courses and SAWB may be beneficial, and because some already used this approach with longer courses. Patients were more averse to SAWB for UTIs owing to concerns that it would lead to recurrence and/or complications and resistant UTIs, but seemed more open to it with longer antibiotic courses or for other infections (seen as less likely to recur). Participants thought that SAWB contradicts the well-known advice to ‘complete the course’ so any change would require evidence and explanation. Participants considered that SAWB could facilitate shared decision making (when appropriate), but requires clear communication and personalisation.

### Strengths and limitations

Clinicians and patients with different characteristics were included, thus exploring multiple relevant perspectives. Interviews were conducted until sufficient data were collected to develop well-supported themes (including supplemental interviews after the preliminary analysis). Multiple, experienced qualitative researchers collected, analysed, and interpreted the data, ensuring that different approaches and interpretations were considered. A transparent record of data collection and analysis was kept and guidelines were followed for qualitative research (see Supplementary Box S3).^20^

Despite efforts to purposefully recruit patients, the study did not receive great interest from patients with simple or few UTIs and males. Understandably, the study interested mostly females who experienced complicated and/or recurrent UTIs. Even when reporting a small number of UTIs in the past year in screening and sampling, during the interviews it often became apparent that patients had experienced complications at some point earlier. Thus, patients’ views were informed by experience of more severe and recurrent UTIs than those of the general population; however, the few participants with simple UTIs raised similar concerns about risks of SAWB. Non-GP clinical views are also underrepresented as interest was mostly received from GPs. Recruitment and topic guides focused on UTIs for which antibiotic courses are already relatively short; recruiting participants without the focus on UTIs would provide more in-depth data about views on SAWB for other infections.

### Comparison with existing literature

Reflected in the findings, a recent meta-ethnography found that patients perceived antibiotics as valuable to treat and prevent UTIs but also as a last resort owing to side effects and resistance.^17^ The tension between perceiving antibiotics as necessary for UTIs and concerns about taking antibiotics was apparent in the data and other studies exploring ways to reduce antibiotics for UTIs; for example, by using delayed or back-up prescriptions^21,22^ or non-antibiotic treatments.^23^ These studies and the present study's data suggest that reducing antibiotic use (for example, through delayed prescriptions, non-antibiotic strategies, and SAWB) appears more acceptable for females with less severe, uncomplicated UTIs, if there was evidence that these approaches are safe, and if the benefits of these approaches were explained. The data in the present study agreed with the findings of the meta-ethnography,^17^ including the impact of UTIs on the whole body, quality of life and activities, and a need for information and cure. This highlights the need for effective (including optimal antibiotic and non-antibiotic) treatments to resolve painful UTI symptoms and prevent long-term, life-changing complications.

The main barriers to implementing SAWB related to beliefs about AMR. The participants correctly described AMR as occurring when pathogens (not human bodies^24^) become resistant to antibiotics, and as propelled by antibiotic use. There appears to be some improvement in public knowledge about AMR and appropriate use of antibiotics,^25^ and a decline in expectations for antibiotics, at least for respiratory tract infections.^26^ Similarly to a systematic review,^24^ in the present study most clinicians and all patients perceived not completing (or too short) antibiotic courses as causing AMR. This is based on a belief, reinforced by public health messages, that if pathogens are not fully eradicated (for example, owing to incomplete or too short antibiotic course), the remaining ones lead to recurrence of resistant infections. However, the threat of ‘target selection’ has been questioned, while the risks of ‘collateral selection’ occurring during antibiotic treatment, and which can be mitigated by shorter exposure to antibiotics, have been highlighted.^7,10^ Many participants stressed the importance of prescribing or taking antibiotics for optimal (that is, minimal effective) duration. A better understanding and evidence is needed on how exactly AMR develops in different infections and how it relates to antibiotic course lengths. Taking antibiotics for optimal, evidence-based durations was a priority over SAWB.

Another difficulty with the SAWB approach related to defining and explaining when exactly antibiotics can and should be stopped. Most participants thought it would be difficult for patients to know exactly at which point in symptom improvement stopping antibiotics was appropriate. Patients suggested that it would be clearer and more appropriate if they were advised to stop antibiotics when completely symptom-free or for a specified time after that. This was seen as possible in UTIs because UTI symptoms were described to appear and cease rapidly; the same might be more difficult for infections in which symptoms improve more gradually. Moreover, quite a few patients wanted to have reliable and accurate tests to ‘objectively’ indicate whether the infection was completely resolved before stopping antibiotics. This fits with other studies showing that point-of-care tests are often seen as ‘objective’ evidence and used as tools to reassure patients of no need for antibiotics.^27,28^

The study found some amenability to the SAWB approach, especially among clinicians, and if the concerns about evidence, safety, and communication were addressed. While many antibiotic-related campaigns still promote the ‘finish your prescription’ messages internationally, the World Health Organization has eliminated this message since 2017.^29^ The present study's findings support the move away from this instruction towards more personalised advice and nuanced understanding of antibiotics and AMR.

### Implications for research and practice

Clinicians and patients expressed the need for evidence on safety and benefits of SAWB, and its impact on AMR, which is the main implication for research. When developing the evidence, it is also important to define the optimal point at which antibiotics should be stopped, that is, after symptoms (and which ones) improve or completely resolve. The main implication for clinicians is that currently there is insufficient evidence and support to recommend SAWB in practice. Both clinicians and patients expressed uncertainty or misunderstandings about how AMR develops and ambiguity about the best ways to communicate SAWB, so future practice and public health education should address these issues. Patients also stressed the need for better diagnostic testing and non-antibiotic treatments for UTIs. Box 1 summarises the implications.

In conclusion, this exploratory study found that patients were less accepting of the approach to SAWB, particularly for UTIs, whereas clinicians were more amenable if it was evidence based. The findings highlight, first, the need for good evidence that this novel approach to self-determining antibiotic duration is safe and beneficial. Second, if evidence based, the reasons for changing the advice from ‘finish antibiotic courses’ to SAWB should be explained to patients and public, and SAWB advice should be given with a clear indication of when exactly to stop antibiotics.

## Data

The dataset relied on in this article is available from the corresponding author on reasonable request. The full dataset is not publicly available to protect participants’ anonymity.
